# Benign Multicystic Peritoneal Mesothelioma: A Rare Tumour of the Abdomen

**DOI:** 10.1155/2015/613148

**Published:** 2015-03-17

**Authors:** Soundappan Somasundaram, Monty Khajanchi, Tejas Vaja, Bhushan Jajoo, Amit Kumar Dey

**Affiliations:** Department of General Surgery, Seth GS Medical College and KEM Hospital, India

## Abstract

Benign multicystic peritoneal mesothelioma: a rare tumor of the abdomen, is a diagnostic dilemma. This report emphasizes the importance of diagnostic laparoscopy in the diagnosis of the tumour.

## 1. Introduction

Benign multicystic peritoneal mesothelioma (BMPM), a rare tumor, occurs mainly in women in their reproductive age [1]. The pathogenesis of BMPM is unclear and a controversy regarding its neoplastic and reactive nature exists. Today approximately 130 cases have been reported all over the world and only around 10 cases have been reported from India. We would like to report this case of BMPM we managed.

## 2. Case Report

A 40-year-old gentleman with no previous medical or surgical history presented with chief complains of vague abdominal pain in right lumbar and iliac fossa. Pain was nonradiating and there was no relation with food intake or with defecation. There was no history of fever or vomiting or similar pain in the past. On examination there was a diffuse nontender lump felt on the right side of the abdomen extending from the right hypochondrium to iliac fossa.

**Figure 1:**
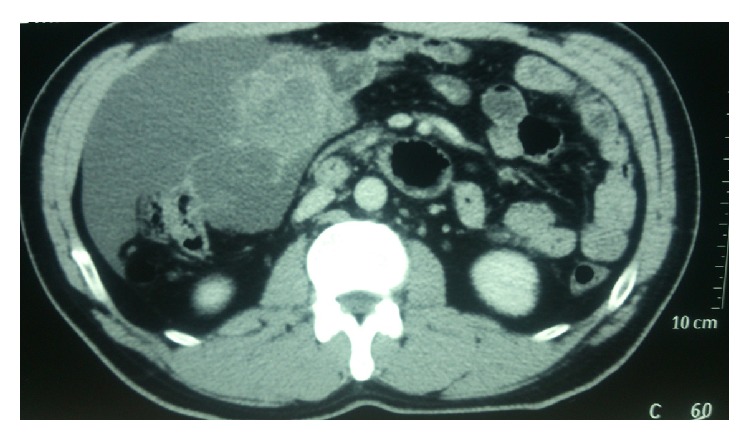
Contrast enhanced computed tomography (CECT) of abdomen and pelvis showing similar size irregular multiseptated multicystic lesion engulfing the ascending colon and caecum. Bowel lumen showed no growth. CECT abdomen showing an irregular multiseptated lesion lying over the ascending colon.

Ultrasonography of abdomen showed 14.5 × 7.5 × 15.9 cm multiseptated lesion with no definite solid component with septations showing minimal vascularity which was mainly venous. Lesion was intraperitoneal and anterior to caecum and terminal ileum. Surface of the liver showed no scalloping. Contrast enhanced computed tomography (CECT) abdomen and pelvis showed similar size irregular multiseptated multicystic lesion engulfing the ascending colon and caecum. Bowel lumen showed no growth (Figure 1). CEA and CA 19-9 were done suspecting adenocarcinoma. Results came back negative. Nothing else was done. At this point the question was how to proceed. We thought about doing one of the following three investigations.

1. Ultrasonography (USG)/CT scan guided needle puncture and aspirate and sending fluid for cytology.
2. Diagnostic laparoscopy.
3. Colonoscopy and biopsy.

This case was discussed with the senior surgical team and a differential diagnosis of appendicular abscess or mucinous cyst adenoma/adenocarcinoma of the appendix was made. Colonoscopic biopsy was not done as there was no growth in bowel wall on CECT. CT scan guided biopsy was not done in view of question of yield of the procedure and tracking of malignant cells in the path. In view of these two differentials and after ruling out other options, diagnostic laparoscopy was planned.

**Figure 2:**
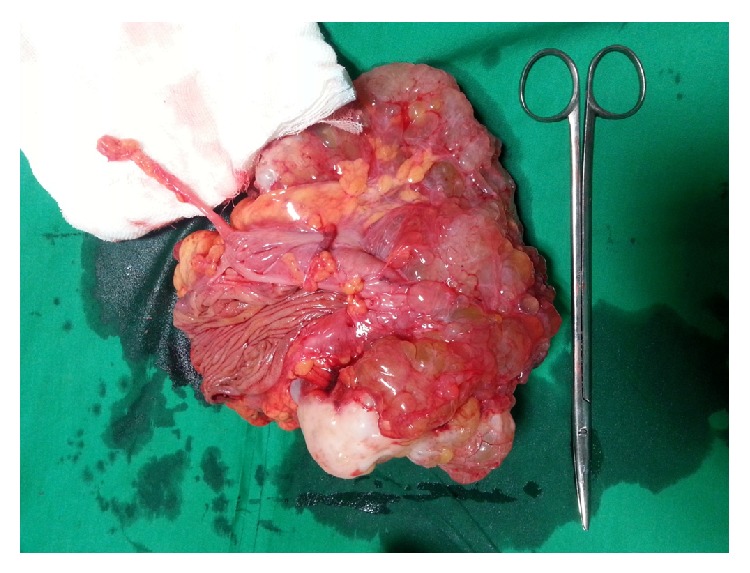
Resected mass with ileocaecum and part of ascending colon. Appendix is also seen. Histopathology on gross examination revealed a cystic mass 21 × 13 × 8 cm attached to the serosa of the caecum without any infiltration into the bowel wall.

Diagnostic laparoscopy revealed multicystic lesion over the whole of the ascending colon and caecum. As the cyst look very fragile, we converted to laparotomy and did a right hemicolectomy with side-to-side ileotransverse anastomosis without causing any rupture of the cyst. Patient had an uneventful postoperative period and was discharged in 8 days (Figure 2).

**Figure 3:**
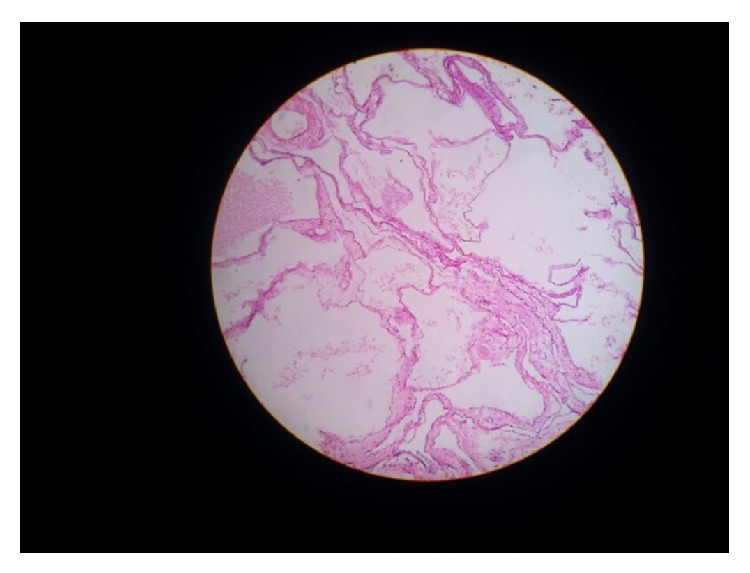
Microscopy showed cuboidal epithelium lined tumor filled with eosinophilic proteinaceous material. Intervening septae showed fibrosis and dense lymphoplasmacytic infiltrate admixed with eosinophils and neutrophils. Microscopic picture of BMPM.

**Figure 4:**
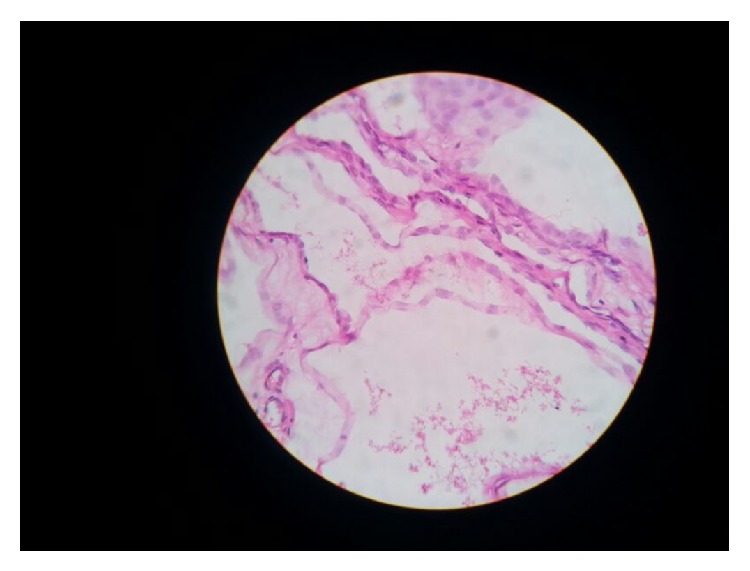
Microscopy shows cuboidal epithelium lined tumor filled with eosinophilic proteinaceous material. Intervening septae showed fibrosis and dense lymphoplasmacytic infiltrate admixed with eosinophils and neutrophils. Microscopic picture of BMPM.

On first follow-up after 1 month patient was well and ultrasonography of abdomen was normal. Histopathology on gross examination revealed a cystic mass of 21 × 13 × 8 cm attached to the serosa of the ceacum without any infiltration into the bowel wall. Cut section showed a multiloculated cyst, each cyst ranging from 0.5 cm to 2.5 cm filled with mucinous material. Lining was smooth and paper thin with few solid areas. Appendix and ascending colon were normal. Proximal and distal resection margins were uninvolved. Microscopy showed cuboidal epithelium lined tumor filled with eosinophilic proteinaceous material. Intervening septae showed fibrosis and dense lymphoplasmacytic infiltrate admixed with eosinophils and neutrophils. No infiltration was seen into the serosa of the caecum. No cytologic atypia or proliferation of the lining epithelium was seen (Figures 3 and 4).

*Immunohistochemistry*. Lining epithelium was positive for calretinin and CD 34 negative.

## 3. Discussion

BMPM was first described in 1979 by Menemeyer and Smith [1]. Since then approximately 130 cases have been reported. BMPM is a localized tumor arising from the epithelial and mesenchymal elements of the mesothelial cells and does not metastasize. It has a strong predilection mostly for the surface of the pelvic viscera and serosal surfaces of the intestine and omentum or in the retroperitoneal space, spleen, and liver [2] our case also showed the origin of the tumor from the serosal surface of the right side colon. The pathogenesis of BMPM is controversial. Some authors believe that the lesion is neoplastic, while others favour a reactive process [3–5]. Some authors have proposed a neoplastic origin based on a slow but progressive growth of the lesions, a tendency to recur after surgical resection, and high disease related mortality in advanced stages. Very rarely malignant transformation takes place [3]. In the literature, only 2 cases have been reported of malignant transformation. BMPM is rarely associated with adenomatoid tumor, another benign mesothelial tumor [4]. Ultrasound and CT do not differentiate benign cystic mesotheliomas from other cystic lesions. In the work-up of cystic peritoneal lesion MRI may confirm the peritoneal origin, differentiate cystic content, or may detect other lesions. In BMPM typical MRI findings are low signal on T1-WI and high signal on T2-WI with peripheral enhancement following Gadolinium administration [5]. While fine-needle aspiration could be used as a diagnostic tool, in most cases this method is not informative. Laparoscopy is the most accurate diagnostic method since it allows visualization, local biopsy of the suspected tissue specimens with intraoperative frozen section if facility permits, though an invasive procedure [6]. There is no consensus on the management protocol. We had chosen diagnostic laparoscopy as there was no clear diagnosis on imaging. Surgery is the only effective treatment for BMPM. Aggressive surgical approaches including cytoreductive surgery with peritonectomy are recommended. Peritonectomy in our kind of case is better to be included in aggressive cytoreductive surgical strategies to avoid local recurrences which are justified through the literature [7, 8]. If a malignant variant is suspected, peritonectomy becomes more necessary which involves a midline laparotomy for maximal visualization of the abdomen and pelvis. Based on the tumour burden in the various regions, a peritoneal cancer index [9] is scored. Once the peritoneal cancer index is scored, cytoreductive surgery is performed. This involves the removal of all visible tumours on the surface of any viscera and the total removal of the diseased peritoneum accordingly [10]. Recurrences occur more frequently in women and treated by hormonal therapy with antioestrogen [8] and gonadotrophin-releasing analogues [8], hyperthermic intraperitoneal chemotherapy using cisplatin or doxorubicin [11], and sclerotherapy with tetracycline. Rosen and Sutton [12] used potassium titanyl phosphate laser to treat these lesions. These methods may be efficacious in the treatment of multiple sites disease and, in addition, may reduce the need for repeated laparotomies. The degree of success following these procedures varies.

## 4. Conclusion

BMPM requires high index of suspicion for diagnosis, so the authors would recommend doing a diagnostic laparoscopy, for all tumors of suspicious etiology on imaging. Diagnosis can only be made on histopathology. Although its recurrence is high after surgical resection, it does not present a tendency to transform into malignancy. Finally, a prolonged systematic follow-up of these patients, perhaps for life, is required since the lesion usually reappears and further resection or another therapy may be indicated.
